# The Tick Microbiome: Why Non-pathogenic Microorganisms Matter in Tick Biology and Pathogen Transmission

**DOI:** 10.3389/fcimb.2017.00236

**Published:** 2017-06-08

**Authors:** Sarah I. Bonnet, Florian Binetruy, Angelica M. Hernández-Jarguín, Olivier Duron

**Affiliations:** ^1^UMR BIPAR INRA-ENVA-ANSESMaisons-Alfort, France; ^2^Laboratoire MIVEGEC (Maladies Infectieuses et Vecteurs: Écologie, Génétique, Évolution et Contrôle), Centre National de la Recherche Scientifique (UMR5290), IRD (UMR224), Université de MontpellierMontpellier, France; ^3^SaBio Instituto de Investigación en Recursos Cinegéticos CSIC-UCLM-JCCMCiudad Real, Spain

**Keywords:** tick, tick symbionts, tick borne pathogens, microbiome, microbial interactions

## Abstract

Ticks are among the most important vectors of pathogens affecting humans and other animals worldwide. They do not only carry pathogens however, as a diverse group of commensal and symbiotic microorganisms are also present in ticks. Unlike pathogens, their biology and their effect on ticks remain largely unexplored, and are in fact often neglected. Nonetheless, they can confer multiple detrimental, neutral, or beneficial effects to their tick hosts, and can play various roles in fitness, nutritional adaptation, development, reproduction, defense against environmental stress, and immunity. Non-pathogenic microorganisms may also play a role in driving transmission of tick-borne pathogens (TBP), with many potential implications for both human and animal health. In addition, the genetic proximity of some pathogens to mutualistic symbionts hosted by ticks is evident when studying phylogenies of several bacterial genera. The best examples are found within members of the *Rickettsia, Francisella*, and *Coxiella* genera: while in medical and veterinary research these bacteria are traditionally recognized as highly virulent vertebrate pathogens, it is now clear to evolutionary ecologists that many (if not most) *Coxiella, Francisella*, and *Rickettsia* bacteria are actually non-pathogenic microorganisms exhibiting alternative lifestyles as mutualistic ticks symbionts. Consequently, ticks represent a compelling yet challenging system in which to study microbiomes and microbial interactions, and to investigate the composition, functional, and ecological implications of bacterial communities. Ultimately, deciphering the relationships between tick microorganisms as well as tick symbiont interactions will garner invaluable information, which may aid in the future development of arthropod pest and vector-borne pathogen transmission control strategies.

## Introduction

Over the last few decades, considerable research efforts have focused on the diversity, distribution, and impact of tick-borne pathogens (TBP). The list of known or potential TBP is constantly evolving, and includes a variety of viruses, bacteria, and parasites afflicting humans and many other animals worldwide (de la Fuente et al., 2008; Heyman et al., 2010; Dantas-Torres et al., 2012; Rizzoli et al., 2014). Less well studied or understood are whole microbial communities hosted by ticks, which attract much less attention, yet are equally important. These communities include TBP, but also non-pathogenic microorganisms such as commensal and mutualistic microbes that are also abundant in ticks (Andreotti et al., 2011; Carpi et al., 2011; Williams-Newkirk et al., 2014; Duron et al., 2015a, 2017). Until recently, these non-pathogenic microorganisms were generally overlooked by scientists working with ticks and TBP. Before 1990, their existence was largely ignored and all bacteria found in ticks were usually considered to be potential TBP without necessarily undergoing rigorous health risk assessment. Toward the end of the 1990s, the advent of simple PCR assays led to a growing understanding that a few intracellular bacteria, such as the *Coxiella*-like endosymbiont and the *Francisella*-like endosymbiont (*Coxiella*-LE and *Francisella*-LE hereafter), are actually non-pathogenic microorganisms hosted by ticks (Niebylski et al., 1997a; Noda et al., 1997). Deeper investigation of microbial biodiversity through high-throughput sequencing and DNA barcoding led to another leap in understanding: non-pathogenic microorganisms from many different clades are present in ticks, and can generally coexist with TBP (Clay et al., 2008; Andreotti et al., 2011; Carpi et al., 2011; Lalzar et al., 2012; Vayssier-Taussat et al., 2013; Qiu et al., 2014; Williams-Newkirk et al., 2014; Narasimhan and Fikrig, 2015; Abraham et al., 2017). In addition, current available data on tick microbiomes suggest that non-pathogenic microorganisms exhibit higher taxonomic diversity than TBP since they encompass most major bacterial and Archaea groups (Andreotti et al., 2011; Carpi et al., 2011; Nakao et al., 2013; Qiu et al., 2014; Williams-Newkirk et al., 2014). Altogether, it is now clear that ticks carry complex microbial communities that are largely dominated by non-pathogenic microorganisms. Most importantly, this implies that both ticks and TBP are commonly engaged in interactions with non-pathogenic microorganisms.

The composition of these microbial communities is highly variable: environmental constraints are key drivers of their structure as shown by differences in bacterial diversity observed between laboratory-reared and wild ticks (Heise et al., 2010; Zolnik et al., 2016). It was further reported that bacterial community structures could vary depending on the examined tick species (Lalzar et al., 2012), the season during which ticks were collected (Lalzar et al., 2012), the examined geographical regions (van Overbeek et al., 2008; Carpi et al., 2011; Williams-Newkirk et al., 2014), the examined tick life stage (Moreno et al., 2006; Clay et al., 2008; Williams-Newkirk et al., 2014; Zolnik et al., 2016), and between different feeding statuses (Heise et al., 2010; Menchaca et al., 2013; Zhang et al., 2014). Furthermore, bacterial community structures may differ depending of the presence of pathogens (Steiner et al., 2008; Abraham et al., 2017). Overall, the quantity of potential variations highlights the lability of microbial communities hosted by ticks, and future studies should focus on understanding how these variations impact tick biology. Below, we will discuss the interesting hypothesis that the inherent flexibility of microbial communities may help ticks adapt to environmental stresses, such as TBP presence.

**Figure 1:**
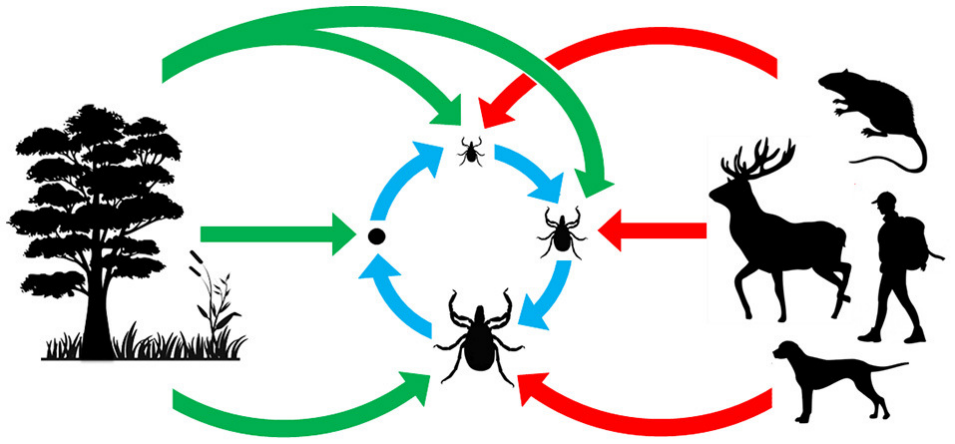
Origin and acquisition of tick microorganisms. Red arrows: vertebrate pathogens acquired from tick bites; blue arrows: maternally inherited tick symbionts acquired via transovarial and transtadial transmission; green arrows: microorganisms acquired from the environment.

Microorganisms inhabiting ticks are not only taxonomically diverse, they are also ecologically diverse. This diversity is clearly illustrated by the large panel of lifestyle strategies that microorganisms use to infect and persist within tick populations (Figure 1). As vertebrate pathogens, TBP normally spread via infectious (horizontal) transmission through tick bite and blood feeding. A few TBP can also be vertically transmitted in ticks, and thus be maintained throughout each generation as observed for *Babesia* species (Chauvin et al., 2009), *Rickettsia rickettsii* (Burgdorfer et al., 1981), or viruses (Xia et al., 2016). Other tick microorganisms are highly specialized intracellular symbionts depending almost exclusively on maternal (transovarial) transmission to ensure their persistence in tick populations (Niebylski et al., 1997b; Lo et al., 2006; Sassera et al., 2006; Klyachko et al., 2007; Felsheim et al., 2009; Machado-Ferreira et al., 2011; Lalzar et al., 2014; Duron et al., 2015a; Kurtti et al., 2015). Tick microorganism diversity is further augmented due to the fact that environmental microorganisms can also colonize ticks: microbes present on vertebrate skin surfaces may colonize ticks during blood feeding, while those present in the soil or vegetation can colonize ticks on the ground, once they have dropped off their vertebrate hosts (Narasimhan and Fikrig, 2015). Overall, the diverse range of microbial lifestyle strategies creates a complex web of interactions offering excellent opportunities to tackle questions about the impact of whole microbial communities on tick biology and TBP transmission. In spite of this, the direct effects of pathogens and other microbes on tick physiology and activity has received much less attention than their effects on vertebrate hosts. In most cases, the function of tick endosymbionts in relation to their host has not been determined. Many of these endosymbionts have obligate intracellular life cycles or are difficult to cultivate, which may explain the gaps in current knowledge (Tully et al., 1995; Kurtti et al., 1996; Niebylski et al., 1997a; Duron et al., 2015a). However, for some bacteria, tissue-specific localization has been defined, which may aid us to understand bacterial impact on both tick biology and pathogen transmission (Noda et al., 1997; Klyachko et al., 2007; Lalzar et al., 2014; Narasimhan and Fikrig, 2015). Similarly, the use of microarray or RNASeq technologies to analyze induced tick microbiome expression patterns and varying composition following a variety of conditions, may also further elucidate their roles (Rodriguez-Valle et al., 2010). This knowledge is of both medical and veterinary interest since it may enable the reassessment of tick-associated health risks, but also of ecological and evolutionary importance by highlighting co-evolutionary processes acting between ticks and their microbes. Indeed, some symbionts, but not all (Weller et al., 1998), have a joint evolutionary history of several million years with their tick hosts (Almeida et al., 2012; Duron et al., 2017), suggesting that complex interactions may have evolved in these associations. If biologists aim to fully understand the ecological and evolutionary processes involved in tick biology and the emergence of tick-borne diseases, a thorough examination of non-pathogenic microorganisms is also required.

Maternally-inherited symbionts are well-known to use specific adaptive strategies to spread and persist within arthropod populations, either providing fitness benefits to female hosts or manipulating host reproduction (Moran et al., 2008; Ferrari and Vavre, 2011). Two categories of widespread endosymbionts are usually recognized in arthropods, although intermediates and transition forms are also frequent:

– The first category consists of obligate (primary) mutualistic symbionts required to support normal host development, thus assisting their host in various essential functions. This includes nutritional diet upgrades by providing biosynthetic pathways absent from their hosts (Moran et al., 2008; Wernegreen, 2012). Indeed, most blood-feeding insects such as bed bugs, lice, and tsetse flies harbor obligate symbionts that provide B vitamins and cofactors not readily obtainable in sufficient quantities from a uniquely blood-based diet (Akman et al., 2002; Hosokawa et al., 2010; Boyd et al., 2013; Nikoh et al., 2014).
– The second category consists of facultative (secondary) symbionts not required for host survival. Some are defensive symbionts conferring protection against natural enemies or heat (Oliver et al., 2010; Ferrari and Vavre, 2011), while others are reproductive parasites that manipulate host reproduction through the induction of parthenogenesis, feminization of genetic males, male-killing, and cytoplasmic incompatibility (conditional sterility between infected and uninfected specimens) (Engelstadter and Hurst, 2009; Cordaux et al., 2011).

In this article we review four major biological aspects where our views on tick microbes have undergone substantial change over the last decade. Firstly, we must emphasize that non-pathogenic microorganisms have much more complex effects on ticks than previously thought. Indeed, it is now evident that several maternally-inherited symbionts are required for tick survival and reproduction, while other symbionts can have multiple effects on tick life history traits. Secondly, whilst tick TBP transmission modes have been studied for decades, we now understand that certain non-pathogenic microorganisms may also interfere with TBP transmission. Thirdly, although microorganisms are often categorized as “TBP,” “commensals,” or “maternally-inherited symbionts,” both intermediate and transitional states frequently occur. In this context, it thus appears vital to not overlook the full range of potential effects, as have been recently described in microbiome studies. And finally, bacterial phylogeny demonstrates that several infectious agents have close genetic proximity with mutualistic tick symbionts. This indicates that some bacterial genera (eg. *Rickettsia, Francisella*, and *Coxiella*) have the capacity to frequently undergo evolutionary shifts between pathogenic and non-pathogenic forms, a process that may lead to the emergence of novel infectious diseases.

## The effect of non-pathogenic microorganisms on tick biology

**Figure 2:**
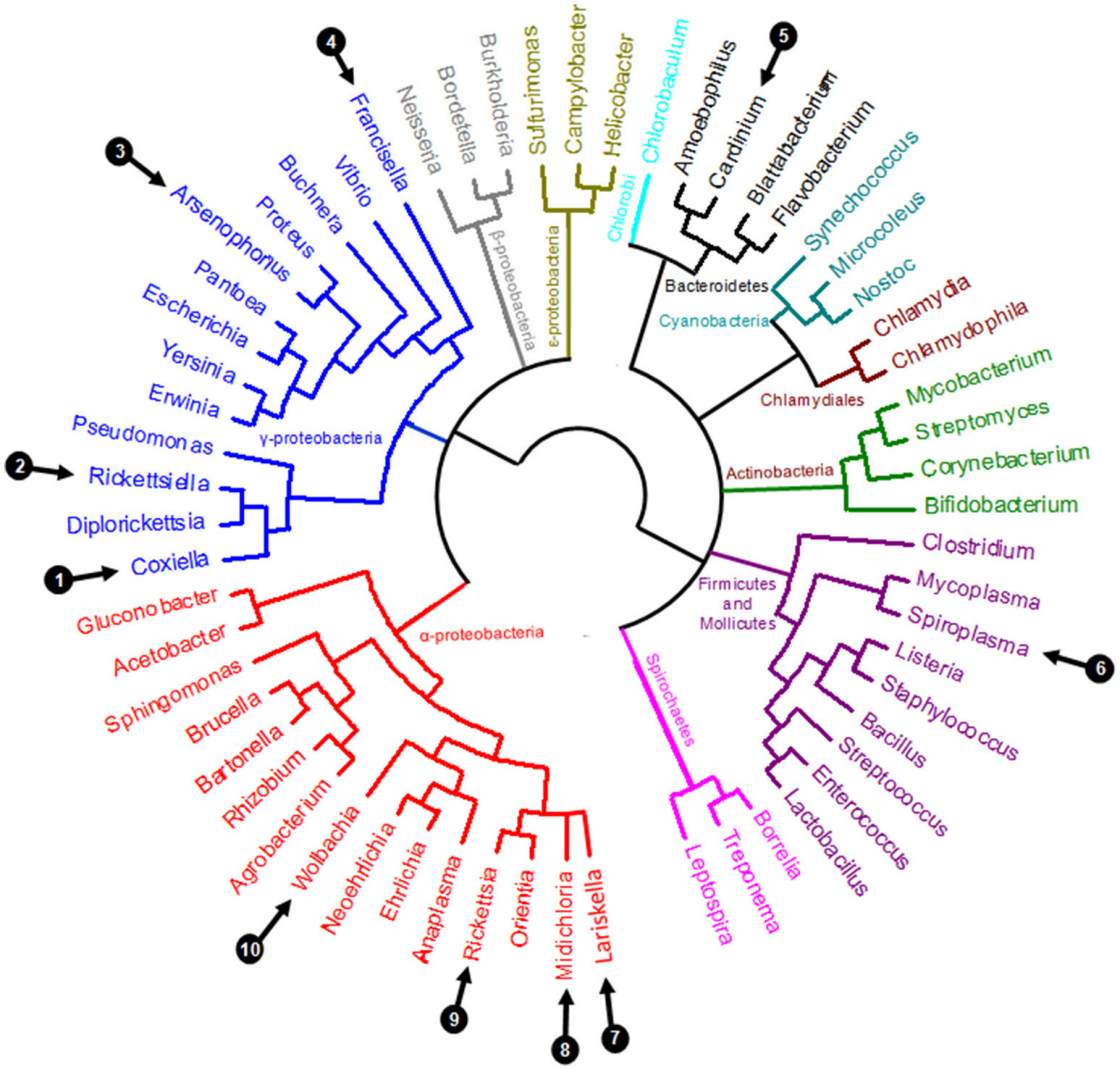
Simplified eubacterial phylogeny showing the evolutionary relationships between the ten genera containing maternally inherited tick symbionts (labeled 1–10, as detailed in Table 1).

Perhaps the most remarkable observation of recent times is the pivotal role of symbiotic interactions in normal tick biology, including ecological specialization to an exclusive blood diet. Symbionts—i.e., microorganisms engaged in close and long-term interactions with their tick hosts—are exceptionally diverse in ticks: at least 10 distinct genera of maternally-inherited bacteria have been reported in ticks over the last decade (listed in Table 1 and Figure 2) (Duron et al., 2017). Three of these symbionts are only found in ticks: *Coxiella*-LE, which infects at least two thirds of tick species, *Midichloria*, which inhabits the mitochondria of some tick species, and *Francisella*-LE, which has only been reported in a few tick species (Table 1). The seven remaining symbiont genera are more- or less-frequently found in other arthropod groups, including several well-studied insects. Five symbionts, *Wolbachia, Cardinium, Arsenophonus, Spiroplasma*, and *Rickettsia*, are commonly identified in some arthropod groups, while two others, *Rickettsiella* and *Lariskella*, have only been reported in a few other arthropod taxa in addition to ticks (Table 1).

**Table 1:** List of the ten maternally inherited bacteria found in ticks and relevant (non-exhaustive) references.

**Maternally inherited bacteria**	**Distribution in ticks and other arthropods**	**Major properties (non-exhaustive references)**
1-*Coxiella*-LE	Very common in ticks, not found in other arthropods (Noda et al., 1997; Jasinskas et al., 2007; Clay et al., 2008; Carpi et al., 2011; Machado-Ferreira et al., 2011; Almeida et al., 2012; Lalzar et al., 2012; Duron et al., 2014, 2015a, 2017)	Obligate symbiont in most tick species (Zhong et al., 2007; Gottlieb et al., 2015; Smith et al., 2015). Closely related to the agent of Q fever, *C. burnetii* (Duron et al., 2015a)
2-*Rickettsiella*	Scattered distribution in arthropods (Tsuchida et al., 2010, 2014; Bouchon et al., 2012; Iasur-Kruh et al., 2013), common in ticks (Kurtti et al., 2002; Vilcins et al., 2009; Anstead and Chilton, 2014; Duron et al., 2015a, 2016, 2017)	Unknown effect in ticks. Facultative mutualist in aphids (Tsuchida et al., 2010, 2014) and likely in other insects (Iasur-Kruh et al., 2013). Some strains are entomopathogenic (Bouchon et al., 2012)
3*-Arsenophonus*	Common in arthropods (Duron et al., 2008a; Novakova et al., 2009), present in ticks (Clay et al., 2008; Dergousoff and Chilton, 2010; Reis et al., 2011; Clayton et al., 2015; Duron et al., 2017)	Male-killer in parasitoid wasps (Werren et al., 1986; Duron et al., 2010), putative obligate symbiont in bat flies and louse flies (Duron et al., 2014), facultative symbiont in other insects (Novakova et al., 2009; Jousselin et al., 2013). In the sheep tick, *Ixodes ricinus*, detection of *Arsenophonus* may be due to contamination by a hymenopteran parasitoid (Bohacsova et al., 2016)
4-*Francisella*-LE	Rare in ticks, not found in other arthropods (Niebylski et al., 1997a; Noda et al., 1997; Scoles, 2004; Goethert and Telford, 2005; Clayton et al., 2015; Gerhart et al., 2016; Duron et al., 2017)	Unknown effect in most cases but alternative obligate symbiont in some tick species (Gerhart et al., 2016; Duron et al., 2017); closely related to the agent of tularaemia (*F. tularensis*) (Sjodin et al., 2012)
5-*Cardinium*	Common in arthropods (Zchori-Fein and Perlman, 2004; Duron et al., 2008a,b), present in ticks (Kurtti et al., 1996; Benson et al., 2004; Duron et al., 2017)	Unknown effect in ticks. Reproductive manipulator in diverse insect species (Engelstadter and Hurst, 2009)
6-*Spiroplasma*	Common in arthropods (Weinert et al., 2007; Duron et al., 2008a), present in ticks (Tully et al., 1981, 1995; Henning et al., 2006; Duron et al., 2017)	Unknown effect in ticks. Male-killer in diverse insect species (Engelstadter and Hurst, 2009)
7-*Lariskella*	Rare and with a scattered distribution in arthropods (Matsuura et al., 2012; Toju et al., 2013), rarely reported in ticks (Qiu et al., 2014; Duron et al., 2017)	Unknown effect
8-*Midichloria*	Present in ticks, not found in other arthropods (Lo et al., 2006; Epis et al., 2008; Venzal et al., 2008; Reis et al., 2011; Najm et al., 2012; Qiu et al., 2014; Williams-Newkirk et al., 2014; Paul et al., 2016; Bonnet et al., 2017; Duron et al., 2017)	Unknown effect; inhabits tick mitochondria (Epis et al., 2013)
9-*Rickettsia*	Common in arthropods (Perlman et al., 2006; Weinert et al., 2009), present in ticks (Niebylski et al., 1997b; Clayton et al., 2015; Kurtti et al., 2015; Duron et al., 2017)	Unknown effect in ticks. Reproductive manipulator in diverse insect species (Engelstadter and Hurst, 2009) and defensive symbiont in other insects (Lukasik et al., 2013) closely related to pathogenic strains, often tick-borne, infecting vertebrates (Perlman et al., 2006; Darby et al., 2007; Weinert et al., 2009)
10-*Wolbachia*	Very common in arthropods (Duron et al., 2008b; Zug and Hammerstein, 2012), present in ticks (Andreotti et al., 2011; Carpi et al., 2011; Reis et al., 2011; Duron et al., 2017)	Unknown effect in ticks. Reproductive manipulation in many arthropods (Engelstadter and Hurst, 2009), facultative mutualist (defensive symbiosis) in others such as mosquitoes (Brownlie and Johnson, 2009; Hamilton and Perlman, 2013), obligate symbiont in bed bugs (Hosokawa et al., 2010; Nikoh et al., 2014). At least in the case of the sheep tick, *Ixodes ricinus*, it has been demonstrated that the detection of *Wolbachia* was due to a contamination by a hymenopteran parasitoid (Plantard et al., 2012)

*Adapted from Duron et al. (2017)*.

*Coxiella*-LE has been reported as essential for tick survival and reproduction in the *Amblyomma americanum* lone star tick (Zhong et al., 2007). As an obligate symbiont, *Coxiella*-LE is, by definition, present in most individuals of a given tick species (Clay et al., 2008; Machado-Ferreira et al., 2011; Lalzar et al., 2012; Duron et al., 2015b, 2017): thus their mutualistic relationship is required for the survival of both organisms. Remarkably, some *Coxiella*-LE may form evolutionarily stable associations with their tick hosts that last for millions of years (Duron et al., 2017). These associations typically exhibit strict co-cladogenesis, resulting in congruent host-symbiont phylogenies as recently observed between members of the *Rhipicephalus* genus and their associated *Coxiella*-LE (Duron et al., 2017). The discovery of *Coxiella-*LE in numerous other tick groups (Jasinskas et al., 2007; Clay et al., 2008; Machado-Ferreira et al., 2011; Almeida et al., 2012; Lalzar et al., 2012; Duron et al., 2014, 2015a, 2017), indicates that it is the most widespread and biologically relevant tick symbiont. An examination of *Coxiella*-LE intra-host localization revealed pronounced tissue tropism in all examined tick species. This symbiont typically infects the ovaries (to ensure maternal transmission) and the distal part of Malpighian tubules, suggesting a possible role in nutrition, osmoregulation, or excretion (Klyachko et al., 2007; Machado-Ferreira et al., 2011; Lalzar et al., 2014). Examination of eggs from several tick species confirmed that *Coxiella*-LE is transmitted to >99% of tick progeny, demonstrating highly efficient maternal transmission (Machado-Ferreira et al., 2011; Lalzar et al., 2014; Duron et al., 2015a). Remarkably, the *Coxiella*-LE genome was shown to encode major B vitamin synthesizing pathways such as biotin (B7 vitamin), folic acid (B9), riboflavin (B2), and their cofactors, that are not usually obtainable in sufficient quantities from a uniquely blood-based diet (Gottlieb et al., 2015; Smith et al., 2015). By ensuring nutritional upgrading of the blood diet, *Coxiella*-LE has enabled ticks to utilize an unbalanced dietary resource and thus become hematophagy specialists.

Recent studies have suggested that alternative obligate symbionts other than *Coxiella*-LE may also exist. Around one third of examined tick species lack *Coxiella*-LE or harbor *Coxiella*-LE at much lower frequencies than expected for an obligate symbiont (Duron et al., 2014, 2015a, 2017). A large survey of 81 tick species showed that in almost all tick species without *Coxiella*-LE infection, another maternally-inherited symbiont was usually present (Duron et al., 2017). Among these alternative obligate symbionts were *Francisella*-LE, *Rickettsia*, and *Rickettsiella*, which are often present in all specimens of the infected tick species (Duron et al., 2017). Although formal testing with nutritional and physiological experiments is now required to validate their role as alternative obligate symbionts, recent bacterial genome data suggest that these bacteria have highly-evolved adaptive mechanisms enabling tick survival. Indeed, their genomes encode functions suggesting that they have—at least partially as for *Coxiella*-LE—a genetic capability for *de novo* B vitamin synthesis. Indeed, the *Francisella*-LE genomes from the fowl tick *Argas persicus* and the Gulf Coast tick *Amblyomma maculatum* contain complete genetic pathways for biotin, folic acid, and riboflavin biosynthesis (Sjodin et al., 2012; Gerhart et al., 2016). Similarly, recent metabolic reconstructions of *Rickettsia* genomes indicated that all genes required for folic acid biosynthesis are present in *Rickettsia* symbiont genomes obtained from both the black-legged tick *Ixodes scapularis* and the Western black-legged tick *I. pacificus* (Hunter et al., 2015). Worthy of note here is that laboratory findings directly corroborate the existence of beneficial *Rickettsia* symbionts since they exert a significant effect on larval motility of *A. americanum, Dermacentor variabilis*, and *I. scapularis* ticks (Kagemann and Clay, 2013). Overall, these maternally-inherited symbionts are thus of ecological and evolutionary importance to the tick species they infect, and potentially mediate tick adaptation to hematophagy. In addition, it should be notice that some tick-borne pathogenic Anaplasmataceae bacteria (including *Anaplasma phagocytophilum, Ehrlichia chaffeensis*, and *Neorickettsia sennetsu*) are also able to synthetize all major vitamins, suggesting that they may also confer a beneficial role in ticks when present (Dunning Hotopp et al., 2006).

Many *Rickettsia* species are well-known TBP, therefore a comment on the true ecological diversity existing within the *Rickettsia* genus is appropriate. Indeed, most of the novel *Rickettsia* species or strains discovered in recent years are found exclusively in arthropods and never in vertebrates (Perlman et al., 2006; Darby et al., 2007; Weinert et al., 2009). In ticks, as for many other arthropods, these *Rickettsia* are not pathogenic but are actually maternally-inherited symbionts with poorly known effects on tick biology. This is the case for the *Rickettsia* species identified in the black-legged tick *I. scapularis* (*Rickettsia buchneri*, formerly known as *Rickettsia* REIS; Kurtti et al., 2015), the American dog tick *Dermacentor variabilis* (*Rickettsia peacockii*; Felsheim et al., 2009), and likely the tree-hole tick *Ixodes arboricola* (*Rickettsia vini*; Duron et al., 2017). The fact that ticks carry both pathogenic and non-pathogenic *Rickettsia* that may interact (as early reported by Burgdorfer et al., 1981), underscores the need to be able to clearly distinguish between the two in further studies on these bacteria.

Along with obligate symbionts, ticks commonly harbor facultative symbionts belonging to a variety of bacterial genera (listed in Table 1). Examination of a representative collection of 81 tick species (i.e., approximately 10% of known tick species and including both soft and hard ticks) illustrated facultative symbiont diversity, since it revealed the presence of maternally-inherited bacteria in almost all species (79 of 81) (Duron et al., 2017). Remarkably, many of these tick species (44) hosted more than one symbiont. In multi-infected tick species, symbionts assembled in communities which could reach high levels of complexity. Indeed, six distinct genera of maternally-inherited symbionts co-exist in sheep tick *I. ricinus* populations (*Midichloria, Spiroplasma, Coxiella*-LE, *Rickettsia, Wolbachia*, and *Rickettsiella*) and in African blue tick *Rhipicephalus decoloratus* populations (*Midichloria, Coxiella*-LE, *Francisella*-LE, *Rickettsia, Cardinium*, and *Spiroplasma*) (Duron et al., 2017). It should be noted that detecting a heritable bacterium can sometimes be due to cross-contamination as for several *I. ricinus* studies. Some recorded *Wolbachia* and *Arsenophonus* infections were actually due to the cryptic presence of a *Wolbachia*- and *Arsenophonus*-infected endoparasitoid wasp, *Ixodiphagus hookeri*, within tick tissues (Plantard et al., 2012; Bohacsova et al., 2016). As a result, the presence of at least some of these tick symbionts must be treated with caution.

Although the role of these facultative symbionts is not yet clearly elucidated, one study suggested that *Arsenophonus* sp. can affect host-seeking success by decreasing *A. americanum, I. scapularis*, and *D. variabilis* tick motility when *Rickettsia* symbionts increased such mobility (Kagemann and Clay, 2013). Recent sequencing and analysis of the *Midichloria mitochondrii* genome led to the hypothesis that the bacteria could serve as an additional ATP source for the host cell during oogenesis (Sassera et al., 2011). In addition, this symbiont has been ascribed a possible helper role in tick molting processes (Zchori-Fein and Bourtzis, 2011). As mentioned above, there is no evidence to date showing that the *Wolbachia* detected in ticks are “true” tick symbionts (Plantard et al., 2012). Interestingly, in insects, *Wolbachia* is known to act as defensive endosymbionts (reviewed by Brownlie and Johnson, 2009), or as manipulator of host reproduction (review in Engelstadter and Hurst, 2009; Cordaux et al., 2011), suggesting that similar effect may exist in ticks.

The same questioning are required regarding *Arsenophonus nasoniae*: known to be responsible for sex-ratio distortion in diverse arthropod species (Werren et al., 1986; Duron et al., 2010) but of unknown effect in ticks. Finally and interestingly, a *Rickettsiella* symbiont has been observed in a parthenogenetic laboratory colony of the tick *I. woodi* (Kurtti et al., 2002). This tick species is generally known to be bisexual, suggesting that *Rickettsiella* infection may induce asexuality which represents to date the only demonstration of sex ratio distortion in ticks possibly due to a symbiont.

High symbiont infection frequency is rarely observed within each tick species, and an intermediate infection frequency is much more common (Noda et al., 1997; Clay et al., 2008; Lalzar et al., 2012; Duron et al., 2017). Interestingly, infection frequencies of each maternally-inherited symbiont are often variable between geographic populations of a given tick species (Clay et al., 2008; Lalzar et al., 2012; Duron et al., 2017). This is the case for the soft tick *Ornithodoros sonrai*, with *Midichloria* and *Rickettsia* reaching high infection frequencies in some populations, but remaining absent from others (Duron et al., 2017). Similar geographical patterns were observed for many other tick species and for several symbionts such as *Rickettsiella* and *Spiroplasma* in the tree-hole tick *I. arboricola* and the polar seabird tick *I. uriae* (Duron et al., 2016, 2017). These patterns strongly suggest that maternally-inherited symbiont infection dynamics are variable across tick populations and species. Such infection frequency variations may be driven by costs and benefits associated with harboring maternally-inherited symbionts, and may maintain intermediate frequencies in tick populations, as is commonly observed in other arthropod species (Oliver et al., 2010). However, even if the nature of these costs and benefits has been well-studied in insects, they remain to be determined in ticks. Another important parameter in our understanding of this infection dynamics may be variation of biological features between tick males and females: indeed, adult males from *Ixodes* species do not blood-feed, contrary to females, which may imply that adult males do not need nutritional symbionts. The importance of this “sex” parameter to symbiont infection dynamics remains also to be demonstrated. Interestingly, this pattern was observed for *Midichloria* with males less commonly infected than females in *I. ricinus* (Lo et al., 2006). This suggests that *Midichloria* may be an important nutritional symbiont for *I. ricinus*, as recently proposed (Duron et al., 2017).

Along with maternally-inherited symbionts, other non-pathogenic microbes are present in ticks (Andreotti et al., 2011; Carpi et al., 2011; Narasimhan et al., 2014; Qiu et al., 2014; Williams-Newkirk et al., 2014; Abraham et al., 2017). Most are likely to inhabit the tick gut, while others may also colonize the tick surface cuticle. Overall, the biological effects of these non-pathogenic microbes on ticks remain entirely unknown, but it is likely that those inhabiting the tick gut could participate in blood meal digestion (Narasimhan and Fikrig, 2015) and complex interactions with TBP as we further detailed.

## Non-pathogenic microorganisms interact with TBP in different ways

An alternative fascinating aspect is that non-pathogenic microorganisms can also interfere with TBP replication and transmission by influencing TBP abundance and diversity in tick populations, as well as their transmission to vertebrate hosts. All aforementioned non-pathogenic bacteria present in ticks could have the potential to impact TBP infection processes in different ways. One can assume that TBP and non-pathogenic microorganisms may neutralize each other because they are in direct competition for limited resources, such as a particular nutrient or tissue, or because they stimulate the same immune system function. Alternatively, non-pathogenic microorganisms may excrete molecules directly inhibiting the growth of a TBP competitor, or, inversely, facilitate TBP development by immunosuppressing the tick host. As a result, non-pathogenic microorganisms may facilitate, limit, or block TBP transmission, depending on the nature of tick microbial interactions.

Equally pertinent in this regard is the role of the microbiota inhabiting the tick gut (Narasimhan et al., 2014; Abraham et al., 2017). The tick gut represents the TBP entry point, therefore gut microbiota can directly mediate TBP colonization and influence their early survival (Narasimhan and Fikrig, 2015). This finding has been perfectly illustrated in a recent study which manipulated the gut microbiota of the black-legged tick *I. scapularis*: specimens reared in sterile containers (i.e., thus preventing preventing external bacterial contamination) showed increased engorgement weights, and decreased colonization by the causative agent of Lyme disease, *Borrelia burgdorferi*, when compared to normal specimens (Narasimhan et al., 2014). Similarly, ticks fed on antibiotic-treated mice exhibited modified gut microbiota that also resulted in increased feeding and low *B. burgdorferi* colonization rates (Narasimhan et al., 2014). Altering the gut microbiota was actually found to decrease production of a glycoprotein from the tick peritrophic matrix, which separates the gut lumen from the epithelium. This peritrophic matrix is pivotal for *Borrelia* colonization success, as it protects *B. burgdorferi* colonizing the gut epithelial cells from toxic gut lumen compounds. Compromised peritrophic matrix due to altered gut microbiota will thus impede *B. burgdorferi* colonization. However, the reverse is true for another TBP, the anaplasmosis agent, *A. phagocytophilum* (Abraham et al., 2017). Remarkably, this bacterium manipulates the gut microbiota of *I. scapularis* to favor its establishment. By inducing tick glycoprotein production, *A. phagocytophilum* partially blocks bacterial biofilm formation, and thus reduces peritrophic matrix integrity, rendering the tick more susceptible to infection (Abraham et al., 2017). Altogether, these observations have uncovered a “Dr. Jekyll and Mr. Hyde”-like role of the tick gut microbiota, so that an unaltered gut microbiota will favor colonization by some TBP, such as *Borrelia*, whereas it may also block colonization by other TBP, such as *Anaplasma*. This antagonistic effect of tick gut microbiota on TBP may explain the rarity of *Borrelia*-*Anaplasma* co-infections in ticks collected from the field (Abraham et al., 2017).

Other interaction mechanisms may also exist. In well-studied animals, such as insects, antagonistic interactions arise when horizontally-transmitted parasites and vertically-transmitted microorganisms co-infecting the same host have conflicting evolutionary interests (Haine et al., 2005; reviewed by Haine, 2008; Ben-Ami et al., 2011; Hamilton and Perlman, 2013). Vertically-transmitted microorganisms, such as maternally-inherited symbionts, are under strong selection pressure to enhance the reproductive success of the hosts they infect (Moran et al., 2008). Conversely, parasites are typically transmitted between unrelated hosts and are therefore not directly affected by altered host fecundity. This conflict of interest has favored the emergence of defensive symbionts in insects, including maternally-inherited symbionts that protect their insect host against a variety of pathogens (reviewed by Haine, 2008; Brownlie and Johnson, 2009). For instance, some maternally-inherited symbionts such as *Wolbachia* may interfere with the replication and transmission of a wide range of pathogens (including viruses, bacteria, protozoa, nematodes, and parasitoids), and protect insects from parasite-induced mortality, possibly by up-regulating the insect's immune system (Brownlie and Johnson, 2009; Gross et al., 2009). Recently, some of these findings have been applied to the development of parasite control methods, where *Wolbachia* infection has been used to limit the vector competence of mosquitoes (Hoffmann et al., 2011). In comparison, very little was known about the existence of defensive symbionts in ticks. However, Burgdorfer et al. were the first to report the presence of defensive symbionts in the Rocky Mountain wood tick *Dermacentor andersoni*. In this tick, a maternally-inherited symbiont, *Rickettsia peacockii*, hampered the multiplication and transovarial transmission of the spotted fever agent, *Rickettsia rickettsii* (Burgdorfer et al., 1981). Similarly, resistance of the ovaries of *D. variabilis* to co-infection with *Ricketisia montana* and *Rickettsia rhipicephali* has been reported (Macaluso et al., 2002). Other experiments on *D. andersoni* further showed that *A. marginale* infection density was negatively correlated to the infection density of another maternally-inherited symbiont, *Rickettsia belli* (Gall et al., 2016). In *I. scapulari*s, it has also been reported that male ticks infected by the maternally-inherited symbionts *Rickettsia buchneri* have significantly lower rates of *B. burgdorferi* infection than symbiont-free males (Steiner et al., 2008). Overall, these observations suggested that the maternally-inherited *Rickettsia* symbionts may be major defensive symbionts protecting ticks against TBP colonization. As a result, *Rickettsia* symbionts may be a key factor influencing TBP abundance and diversity in tick populations.

Conversely, maternally-inherited symbionts may not always protect ticks against pathogens: the presence of one maternally-inherited symbiont, *Francisella*-LE, in *D. andersoni* was positively associated with pathogenic *Francisella novicida* infection (Gall et al., 2016). Because these results were only obtained following laboratory manipulations, they should be treated with caution as *F. novicida* is not considered to be a TBP, as the majority of people infected with *F. novicida* contract the pathogen after ingesting infected water or ice, and not via tick bites. This study thus relies on an artificial *F. novicida* tick infection that is unlikely to happen in the field, and most importantly, using a pathogen that has not co-evolved with tick symbionts. This naturally raises the question of whether *Francisella*-LE can actually protect *D. andersoni* against TBPs that naturally occur in this tick species.

## Tick symbionts can be opportunistic vertebrate pathogens

Although biologists often classify host-microbe relationships as either “mutualism,” “commensalism,” or “parasitism,” there are difficulties in defining the boundaries of these definitions. Rather, host-microbe relationships should be best described as a broad continuum, as intermediate states and transitions between states occur frequently. Several maternally-inherited tick symbionts are remarkable examples of this continuum, as recent literature has reported that certain symbionts may be transmitted to vertebrates following tick bite, as will be detailed further in this section (Shivaprasad et al., 2008; Woc-Colburn et al., 2008; Vapniarsky et al., 2012; Bazzocchi et al., 2013; Edouard et al., 2013; Angelakis et al., 2016; Seo et al., 2016a; Bonnet et al., 2017). Most importantly, some of these symbionts have the potential to opportunistically infect vertebrate hosts, including humans.

Maternally-inherited arthropod symbionts are commonly thought to be exclusively domesticated by their arthropod hosts: they cannot invade naïve hosts and have evolved to be dependent on arthropod-based transmission mechanisms through transovarial inheritance (Moran et al., 2008; Wernegreen, 2012). However, some tick symbionts, such as certain *Coxiella, Midichloria* and *Arsenophonus* strains, are not actually completely dependent on ticks. Rather than strictly maternal, their transmission may be partially horizontal, i.e., infectious, thus presenting a substantial infection risk to vertebrates (Shivaprasad et al., 2008; Woc-Colburn et al., 2008; Vapniarsky et al., 2012; Bazzocchi et al., 2013; Edouard et al., 2013; Angelakis et al., 2016; Seo et al., 2016a). Among these symbionts, *Coxiella*-LE are the most commonly found microorganisms in vertebrates. Indeed, tick-transmitted *Coxiella*-LE has recently been reported to cause mild infectious symptoms in humans from Europe (Angelakis et al., 2016). These microorganisms were notably detected in human skin biopsy samples and may be a common causative agent of scalp eschar and neck lymphadenopathy. *Coxiella*-LE infections have also been occasionally reported in pet birds such as psittacines and toucans reared in North America (Shivaprasad et al., 2008; Woc-Colburn et al., 2008; Vapniarsky et al., 2012). These latter *Coxiella*-LE can cause fatal disease: infected birds exhibited lethargy, weakness, emaciation, and progressive neurologic signs for several days prior to death. Conversely, another *Coxiella*-LE was identified in several South Korean horse blood samples, but none of the horses showed apparent symptoms of infection (Seo et al., 2016b).

The ability of *Coxiella*-LE to infect vertebrates through tick biting is at least partially explained by their tissue tropism within the tick body. Aside from tick ovaries and Malpighian tubules, examination of tick internal organs also revealed substantial *Coxiella*-LE concentrations within the salivary glands of some tick species (Klyachko et al., 2007; Machado-Ferreira et al., 2011; Qiu et al., 2014) but not in others (Liu et al., 2013; Lalzar et al., 2014). This tissue tropism may enable *Coxiella*-LE release into the vertebrate during tick bite, thus favoring opportunistic infections (Duron et al., 2015a). The overall likelihood of such *Coxiella*-LE tick-to-vertebrate transfers seems high since (i) ticks are found worldwide and feed on many different vertebrate species, (ii) at least two thirds of tick species are infected by *Coxiella*-LE, and (iii) when present in a given tick species, *Coxiella*-LE are usually present in almost all specimens (Duron et al., 2015a). Overall, these observations suggest that, through tick parasitism, vertebrates are often exposed to *Coxiella*-LE, and probably at a higher rate than TBP. However, despite this, *Coxiella*-LE infections are very rare in vertebrates, and most strains described to date have only been identified from ticks (Duron et al., 2015a). It is thus thought that these bacteria pose a low infection risk to vertebrates because their genome seems to be extremely reduced and is devoid of known virulence genes (Gottlieb et al., 2015; Smith et al., 2015). Nonetheless, *Coxiella*-LE have the potential to cause rare infections in vertebrates and should always be considered in future studies on tick-borne diseases.

In comparison, vertebrate infections by symbionts other than *Coxiella*-LE are clearly less common. This includes the maternally-inherited symbiont *Arsenophonus*, present in approximately 5% of terrestrial arthropods, including some tick species (Duron et al., 2008a, 2017). *Arsenophonus* is actually unique among maternally-inherited symbionts because it is able to grow outside arthropod cells, in extracellular environments (Huger et al., 1985; Werren et al., 1986). This ability enhances the likelihood of successful opportunistic *Arsenophonus* infection, as was recently observed in a woman who was bitten by a tick during a trip to Southeast Asia. This patient presenting with a rash and an eschar developed a co-infection with *Arsenophonus* and *Orientia tsutsugamushi* (the causative agent of scrub typhus) (Edouard et al., 2013). In this context, it is likely that rash and eschar development following *Orientia* infection may have favored a secondary, opportunistic, *Arsenophonus* infection.

In other cases, the identification of symbionts as opportunistic vertebrate pathogens is more difficult and remains speculative. This is the case for *Midichloria*, an intra-mitochondrial symbiont of the sheep tick *I. ricinus* and a few other tick species. Several lines of evidence have recently suggested that vertebrate hosts can be inoculated with *Midichloria* during a tick bite. Indeed, most *Midichloria* are localized in the tick ovaries, where they are transmitted to the progeny, but some have also been detected in the salivary glands and saliva of *I. ricinus* (Di Venere et al., 2015). In addition, *Midichloria* DNA, as well as antibodies against a *Midichloria* antigen, were detected in the blood of vertebrates exposed to tick bites (Bazzocchi et al., 2013). However, whether *Midichloria* can cause a true infection and pathological alteration in mammalian hosts remains to be determined.

## Pathogens and tick symbionts are often phylogenetically related

The vast range of intracellular bacteria in ticks is particularly illustrative of their propensity to evolve extreme and contrasting phenotypes. Certain species, such as *Rickettsia* spp. and *Coxiella* spp., have taken eukaryote associations to the extreme by completely abandoning any semblance of a free-living phase and replicating solely within the host cell. However, they do use a large panel of lifestyle strategies to spread and persist within host populations: while some are extremely virulent pathogens, others behave as subtle mutualistic symbionts (Perlman et al., 2006; Darby et al., 2007; Weinert et al., 2009; Sjodin et al., 2012; Duron et al., 2015a, 2017; Gerhart et al., 2016). Although both strategies require high degrees of lifestyle specialization, they are not fixed endpoints along the bacterium-eukaryote interaction spectrum; rather, parasitism and mutualism may shift through repeated evolutionary transitions. This explains why both pathogenic and mutualistic forms of several major bacterial genera commonly hosted by ticks are abundantly represented.

The foremost examples of these transitions are found in three major intracellular bacteria genera: *Coxiella, Francisella*, and *Rickettsia* (Figure 2), which are all commonly identified in ticks. In medical and veterinary research, these intracellular bacteria are traditionally recognized as highly virulent vertebrate pathogens, as they have evolved specific mechanisms to penetrate into the host cytosol, appropriate nutrients for replication, subvert host immune responses, and ultimately enable infectious transmission to a new host individual (Darby et al., 2007; Celli and Zahrt, 2013; van Schaik et al., 2013; Jones et al., 2014). In humans, major intracellular pathogens have been identified from these bacterial groups, as exemplified by the agent of Q fever, *Coxiella burnetii*, the agent of tularaemia, *Francisella tularensis*, the agent of epidemic typhus, *Rickettsia prowazekii*, the agent of Rocky Mountain spotted fever, *Rickettsia rickettsia*, or the causative agent of Mediterranean spotted fever, *Rickettsia conorii* (Figure 2). All of these organisms are extremely infectious and some are currently classified as potential weapons for biological warfare (Darby et al., 2007; Celli and Zahrt, 2013; van Schaik et al., 2013; Jones et al., 2014). In addition, several species of tick-borne bacteria as typified by rickettsiae that were considered non-pathogenic for decades are now associated with human infections (Parola et al., 2013; Bonnet et al., 2017). However, as we have detailed above, novel intracellular bacteria engaged in endosymbiotic associations with arthropod hosts have also recently been discovered within each of these groups (Figure 2).

**Figure 3:**
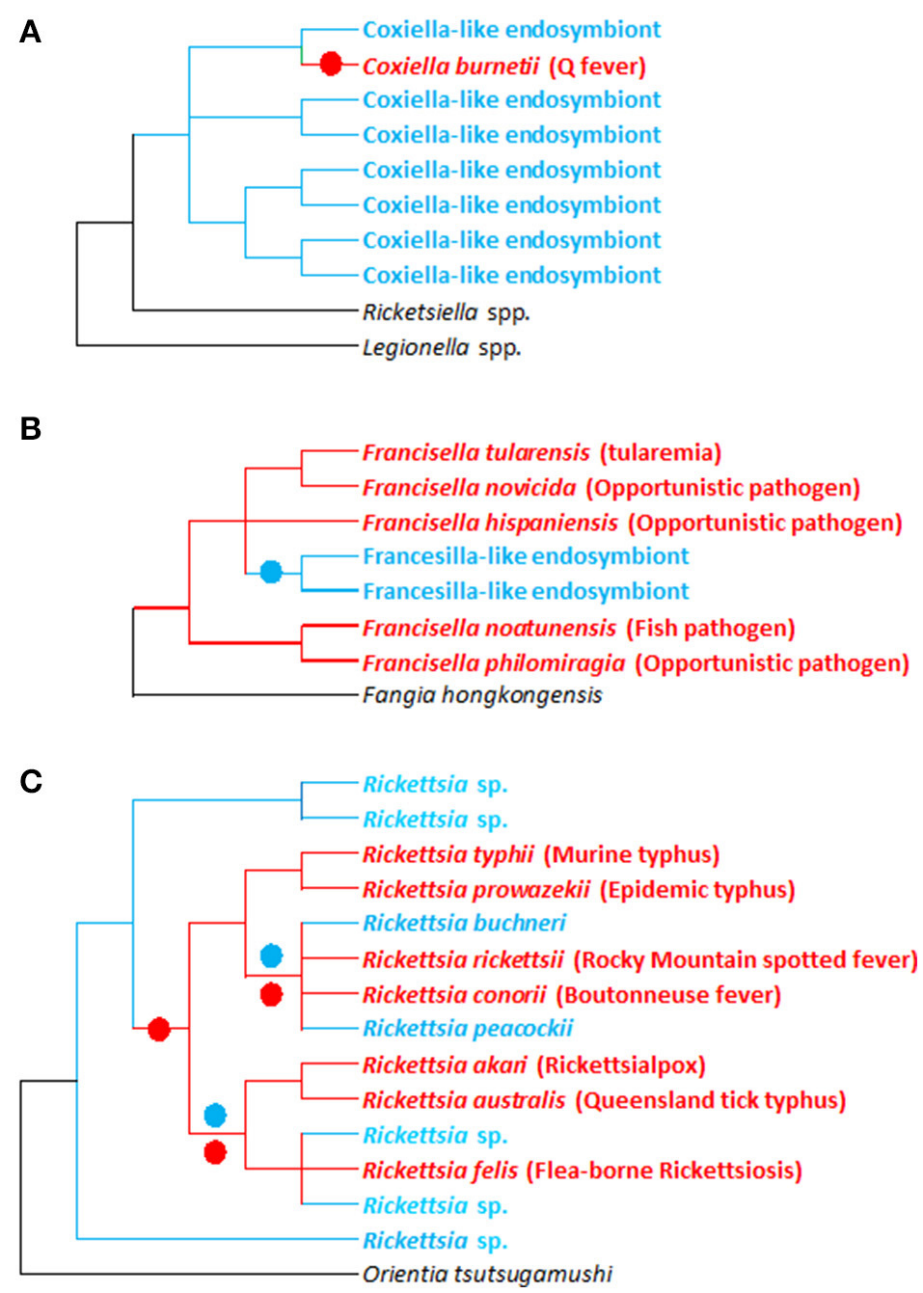
Evolutionary relationships between pathogenic and non-pathogenic (symbiotic) forms within the *Francisella*, *Coxiellai*, and *Rickettsia* bacterial genera. **(A–C)** Simplified phylogenies of *Coxiella, Francisella*, and *Rickettsia*, respectively, adapted from Perlman et al. (2006), Weinert et al. (2009), Duron et al. (2015a), and Sjodin et al. (2012). Red: pathogenic forms; blue: endosymbiotic forms associated with arthropods (ticks for *Francisella* and *Coxiella*; ticks and other arthropods for *Rickettsia*); black: bacterial outgroups. Colored circles on tree branches indicate major evolutionary transitions from symbiotic ancestors to pathogenic descendants (red circles) and from pathogenic ancestors to symbiotic descendants (blue circles).

Phylogenetic investigations have revealed rapid and repeated evolutionary shifts within these three genera between pathogenic (associated with vertebrates and, in some cases, vectored by arthropods) and endosymbiotic forms (specifically linked to arthropods). However, the evolutionary shifting pattern varies among genera (Figure 2). In *Coxiella*, complementary lines of argument indicate a recent emergence of the Q fever agent, *C. burnetii*, from a *Coxiella*-LE strain hosted by soft ticks (Duron et al., 2015a). The *Coxiella* genus displays extensive genetic diversity, with at least four highly divergent clades (Duron et al., 2015a). While *Coxiella*-LE strains hosted by ticks are found in all these clades, all *C. burnetii* strains cluster within one of these clades, delineating an embedded group among soft tick *Coxiella*-LE (Figure 3A). This phylogenetic pattern indicates that the ancestor of *C. burnetii* was a tick-associated *Coxiella* which succeeded in infecting vertebrate cells (Duron et al., 2015a). The remarkably low genetic diversity of *C. burnetii* indicates unique and recent emergence of this highly infectious vertebrate pathogen (Duron et al., 2015a). Interestingly, this hypothesis was initially raised a decade ago from observations of the profound differences in *C. burnetii* genome content relative to other pathogenic intracellular bacteria (Seshadri et al., 2003).

Similarly, in *Rickettsia* spp., recent evidence revealed that human pathogens—vectored by blood feeding arthropods such as ticks—emerged relatively late in the evolution of this genus (Figure 3C; Perlman et al., 2006; Darby et al., 2007; Weinert et al., 2009). Phylogenetic investigations taking into account the entire *Rickettsial* diversity (i.e., including pathogenic and non-pathogenic forms) clearly indicate that switching between hosts (invertebrates, vertebrates, and even plants) has been a common feature of *Rickettsia* evolution (Perlman et al., 2006; Darby et al., 2007; Weinert et al., 2009). Based on current data, it is difficult to estimate how often vertebrate pathogenesis has evolved within *Rickettsia*. But as intracellular adaptation to arthropods is a feature of all current *Rickettsia*, it suggests that their most common recent ancestor was adapted to arthropod endosymbiosis. Surprisingly, comparing human pathogens with closely related non-pathogens showed no relationships between pathogenicity and the acquisition of novel virulence genes: vertebrate virulence seems to occur rather as result of lost or malfunctioning replication systems (Darby et al., 2007).

Conversely, in *Francisella*, the evolutionary pattern is substantially different, since most of the diversity found in this genus is due to pathogenic or opportunistic species (Sjodin et al., 2012). Very little is known about the evolution and origin of tick *Francisella*-LE (Michelet et al., 2013; Gerhart et al., 2016; Duron et al., 2017). However, the few *Francisella*-LE species identified to date delineate a unique monophyletic clade that clearly originates from pathogenic forms (Figure 3B; Duron et al., 2017). Interestingly, the *Francisella*-LE genome is similar in size to pathogenic *Francisella* species' genomes, but about one-third of the protein-coding genes are pseudogenized and are likely non-functional (Gerhart et al., 2016). This suggests that *Francisella*-LE is undergoing a global process of genome reduction, an evolutionary development typically observed in maternally-inherited symbionts (Moran et al., 2008). Interestingly, *Francisella*-LE has conserved intact most of its genes involved in B vitamin biosynthesis, highlighting the pivotal role these genes play in adaption to its current endosymbiotic lifestyle (Gerhart et al., 2016).

Overall, these two very different phenotypes (symbiosis vs. pathogenesis), along with two contrasting transmission modes (vertical vs. horizontal), and variable host specificity (ticks vs. vertebrates), make the *Coxiella, Francisella*, and *Rickettsia* genera especially fascinating. They thus offer an unusual opportunity to answer questions about the origins and mechanisms of symbiosis and pathogenesis. Further studies characterizing host range and infectivity of different genera members would be invaluable to obtaining such results, as would the characterization of tick symbiotic strain genomes. However, research efforts to date have invariably tended to concentrate on their medically important relatives, and so we know comparatively little about the biology of maternally-inherited symbionts. This neglect is unfortunate because fully understanding the whole scope of *Coxiella, Francisella*, and *Rickettsia* phenotypes linked to genome sequences, will provide an excellent system to test hypotheses on the importance of genome content and plasticity in the emergence and reversibility of extreme phenotypes such as symbiosis and pathogenesis.

## Conclusion and perspectives

Extensive literature studies have now made it clear that TBP are not alone: an appreciable range of diverse non-pathogenic microorganisms has also been detected in almost all tick species examined so far. Perhaps the most important consideration for the future is not the incidence of these non-pathogenic microorganisms, but their phenotypes. The varied collection of non-pathogenic microorganisms includes intracellular maternally-inherited symbionts and microbes inhabiting the tick gut, and each could strongly influence—in very different ways—the biology of their tick hosts as well as TBP infection dynamics. Recent findings have shown that several maternally-inherited symbionts such as *Coxiella*-LE are important drivers of evolutionary change in ticks, as clearly shown by their role in driving their tick hosts to adapt to a strict hematophagous diet (Gottlieb et al., 2015; Smith et al., 2015; Duron et al., 2017). Other non-pathogenic microorganisms such as maternally-inherited *Rickettsia* symbionts and gut microbiota are also likely to substantially contribute to the acquisition of ecologically important traits, such as TBP resistance (Burgdorfer et al., 1981; Steiner et al., 2008; Narasimhan et al., 2014; Abraham et al., 2017). It is therefore vital to establish the nature of the interactions between non-pathogenic microorganisms, their tick hosts, and co-infecting TBP. To achieve this goal, it is essential to understand how ticks acquire their microbiota, and how microbial community structures are shaped by various environmental and host factors, and also by microbial interactions within these communities. This knowledge is a key step toward using non-pathogenic microorganisms to limit TBP transmission and persistence. Similarly, whether tick symbionts have the potential to opportunistically infect humans and other vertebrates should be investigated in depth. Lastly, we would like to emphasize that the study of some non-pathogenic microorganisms, such as members of the *Coxiella, Francisella*, and *Rickettsia* genera, can advance our understanding of many infectious diseases including Q fever, tularemia, and rickettsial diseases. The broad phenotypic diversity present in these three bacterial genera make them perfect models to study the evolutionary emergence of pathogenicity and adaptations to living in vertebrate cells. Owing to their medical importance, the pathogenic species of these genera have been the targets of several genome sequencing projects, which have provided insights into the mechanisms and consequences of their specialized lifestyles (Darby et al., 2007; van Schaik et al., 2013). Conversely, the symbiotic forms adapted to tick hosts have received much less attention and a lot of things remain to be elucidated (but see Gillespie et al., 2012, 2015; Clark et al., 2015 for genomics insights about *Rickettsia*). In this context, comparative genomic approaches will be highly valuable in enhancing our understanding of the evolutionary ecology of both pathogenic and non-pathogenic intracellular bacteria, and in identifying novel candidate genes contributing to virulence and persistence in vertebrate cells.

## Author contributions

SB, AH, FB, and OD conducted the literature research, wrote the paper and prepared the figures and tables. All authors provided critical review and revisions.

### Conflict of interest statement

The authors declare that the research was conducted in the absence of any commercial or financial relationships that could be construed as a potential conflict of interest.
